# Health Information Technologies in the Support System of New Fathers’ Postnatal Depression: Scoping Review

**DOI:** 10.2196/95238

**Published:** 2026-08-26

**Authors:** Weijie Wang, Erika Penney, Jaime Garcia

**Affiliations:** 1 Faculty of Engineering and Information Technology, University of Technology Sydney, 15 Broadway, Ultimo, New South Wales, 2007, Australia, 61 0411278545; 2 Faculty of Health, University of Technology Sydney, Ultimo, New South Wales, Australia

**Keywords:** paternal postnatal depression, paternal mental health, digital intervention, digital health, perinatal education

## Abstract

**Background:**

Perinatal mental health issues are widely recognized as a significant public health concern; however, fathers remain underrecognized and underserved within perinatal care systems. Existing reviews have largely focused on mothers, infants, couples, or general paternal mental health interventions, leaving limited synthesis of technology-based and digitally delivered support relevant to paternal perinatal depression. Digital interventions, including SMS programs, online groups, web-based platforms, mobile health apps, and hybrid digital follow-up, may offer flexible ways of engaging fathers; however, their evidence base remains unclear.

**Objective:**

This scoping review aimed to map the available evidence on technology-based, digitally delivered, and hybrid interventions relevant to paternal postnatal depression and paternal perinatal mental health. Specifically, it examined the types of evidence available; how interventions varied by delivery mode, methodology, and population; how interventions were characterized in terms of format and duration; and what outcomes had been measured across studies.

**Methods:**

This review was conducted in accordance with the PRISMA-ScR (Preferred Reporting Items for Systematic Reviews and Meta-Analyses Extension for Scoping Reviews) framework. A comprehensive search was conducted between February and April 2025 and updated in June 2026 across multidisciplinary electronic databases. Search terms covered 4 concepts: fathers or nonbirthing parents, perinatal mental health, digital or technology-based modalities, and intervention or evaluation purpose. Eligible studies were peer-reviewed English-language articles in which fathers, expectant fathers, new fathers, male partners, or nonbirthing parents were active participants, target users, or reported subgroups, and in which a digital, technology-based, or hybrid support strategy was described or evaluated. Data were charted and synthesized descriptively and comparatively.

**Results:**

Six studies met the inclusion criteria. The evidence included a feasibility study of an SMS-based, father-specific program, randomized controlled trials of hybrid psychoeducational or paternal support interventions, a pilot randomized feasibility trial of an online cognitive behavioral therapy–based intervention for pregnant people and partners, a mixed methods online group intervention for new fathers, and a feasibility, acceptability, and usability study of a father-specific mobile health app. Delivery modes included SMS messaging, WhatsApp follow-up, online video, telephone follow-up, online groups, web-based intervention content, and mobile app features. Outcomes included depression, anxiety, stress, parenting stress, dyadic adjustment, marital quality, resilience, usability, acceptability, engagement, adherence, and qualitative user experience. Findings were most consistent for feasibility, acceptability, and engagement, while evidence for effectiveness in reducing paternal postnatal depression remained limited and mixed.

**Conclusions:**

The current evidence base is small, heterogeneous, and preliminary. Digital interventions may offer acceptable ways to engage some fathers and partners during the perinatal period. However, there is insufficient evidence to draw firm conclusions about effectiveness, scalability, or suitability for fathers experiencing perinatal depression. Future research should prioritize father-specific, theory-informed, co-designed digital interventions using validated paternal outcomes, longer follow-up, transparent engagement reporting, and more diverse samples.

## Introduction

The transition to fatherhood can be stressful and overwhelming. Along with the usual difficulties of new caregiver responsibilities, new fathers may face changes to their self-identities, their connection to their partners, and financial commitments [1]. Approximately 10% of new fathers experience depression or anxiety in the perinatal period, and paternal depression has been associated with reduced father involvement and poorer child development outcomes. Decreased paternal engagement in child-rearing can also lead to an increase in children with psychiatric disorders, behavioral issues, and lower academic achievement [2]. Despite this, most perinatal support is received by mothers and infants. Father-specific support is limited, and fathers are often treated as visitors before, during, and after the birth of their baby [3]. Due to stigma and societal perspectives on masculinity, fathers can tend to see themselves in a traditional provider, supporter, and caregiver role, which may create an invisible barrier to seeking emotional support from others [4].

Importantly, unlike the typical signs of mothers’ depression, such as sadness, tearfulness, and withdrawal, new fathers are more likely to present with exhaustion, difficulty focusing, and irritability [5]. These gender differences may contribute to systematic underrecognition of paternal mental health problems. The lack of support can escalate challenges into more acute mental health issues, and fathers’ mental well-being can impact the well-being of partners and children [6].

Existing reviews demonstrate the broader problem but do not yet provide a clear map of appropriate support for fathers’ perinatal depression. Reviews of partner-inclusive or paternal perinatal interventions have found promising effects, but studies often involve mothers, infants, couples, or general parenting support rather than interventions designed primarily for the unique needs of fathers [7-10]. Reviews of paternal perinatal depression and anxiety consistently identify limited father-specific evidence, inconsistent outcome measures, and methodological weaknesses, but have stopped short of proposing a research agenda to overcome them [1,10]. While fathers have been included in some technology-based parenting programs, relatively few have focused on fathers as participants or as primary users of perinatal mental health support. This is significant because technology-based interventions may be well-suited to address the access barriers of fathers.

Digital and technology-based interventions may offer a relevant pathway for engaging fathers during the perinatal period; however, their efficacy and acceptability for this population remain understudied. Digital or technology-based interventions refer to interventions, support strategies, or service pathways in which information or communication technologies are used to deliver, reinforce, monitor, or facilitate mental health support. These may include messaging platforms, mobile health (mHealth) apps, web-based platforms, online groups, online videos, telephone or app-based follow-up, and gamified or game-informed tools.

These modalities may be particularly relevant, given that research has identified barriers to paternal help-seeking, including stigma, masculine norms, limited father-specific services, work and caregiving commitments, and reluctance to engage with conventional face-to-face support [4], highlighting the need for novel interventions to reach this population. Digital technologies may offer this novel, flexible, and private access to support [11], such as father-specific information aligned with perinatal milestones, self-care needs, supporting a partner, and caring for an infant. While digital parenting and online psychological interventions have been increasingly explored in perinatal contexts, fathers are often included as secondary participants rather than primary users [12,13]. Father-specific digital interventions may therefore provide flexible, private, timely, or context-specific support.

Existing perinatal reviews have focused almost exclusively on maternal and infant mental health, systematically excluding fathers as a population of concern. This leaves 2 critical gaps in existing literature. First, fathers are routinely excluded from perinatal interventions, which are primarily designed around mothers’ experiences and needs, resulting in a scarcity of interventions specifically targeting paternal perinatal mental health. Second, the potential of digital delivery modalities to reach this underserved population remains unexplored and unmapped. In this review, the perinatal period is understood as the period from pregnancy through to the first 12 months after birth, which is commonly used in perinatal mental health research and reporting [14], and reflects the psychological continuity between pregnancy, birth, and the postpartum year.

This review aims to map and chart the extent of available evidence for technology-based and digitally delivered interventions for paternal perinatal mental health. The review addresses 3 research questions (RQs). First, what types of evidence exist and how do studies vary by delivery mode, methodology, and population? Second, how are the interventions characterized in terms of delivery mode, format, durations, and intervention components? Third, what outcomes have been measured across studies and what gaps remain in the evidence base?

## Methods

### Overview

This scoping review was conducted in accordance with the latest PRISMA-ScR (Preferred Reporting Items for Systematic Reviews and Meta-Analyses Extension for Scoping Reviews) guidelines (Checklist 1) [15]. The PRISMA-ScR checklist includes 20 essential reporting items and 2 optional items designed to ensure transparency and rigor in scoping review methodology. A comprehensive literature search was conducted between February and April 2025, and updated in June 2026, across 9 electronic databases. The screening process was conducted in 3 stages: title screening, abstract screening, and full-text screening. If eligibility could not be confidently determined at the title or abstract stage, the paper was advanced to the next stage for further review. Papers were assessed to ensure that they met all 5 inclusion criteria. Discrepancies or uncertainties during screening were resolved in discussion with a third reviewer, and consensus was reached in all cases after this discussion.

### Protocol and Registration

This scoping review was conducted in accordance with the PRISMA-ScR framework. A protocol for this review was not prospectively registered; however, the review questions, eligibility criteria, search concepts, and data-charting categories were developed before data extraction to minimize bias.

### Search Strategy and Study Selection

A scoping review format was selected in response to the limited research literature on digital interventions for paternal perinatal mental health. This approach enabled the timely integration of current evidence, highlighted gaps in the literature, and identified key areas for future research.

The search was conducted between February and April 2025 across 9 electronic databases, including technical databases (eg, ACM and IEEE Xplore), Scopus databases (eg, MEDLINE and PubMed), ScienceDirect, SpringerLink, and Wiley. An updated search was conducted in June 2026 to capture recent literature, including an additional search of the PsycINFO database. The search strategy was organized around 4 core concepts: population, mental health or well-being, technology modality, and intervention or evaluation purpose. Search terms included father-related terms, such as father*, paternal, new dad, expecting father, father-to-be, and non-birthing parent*; mental health and well-being terms, such as postnatal depression (PND), postpartum depression, perinatal mental health, anxiety, stress, psychological well-being, adjustment, help-seeking, and psychosocial support; technology terms, such as digital, online, web-based, internet, eHealth, mHealth, mobile app*, smartphone, telehealth, gamif*, game*; and intervention or evaluation terms, such as intervention, program, therap*, treatment, support, education, psychoeducation, feasibility, acceptability, usability, implementation, and evaluation. The query terms used in the search strategy are presented in Textbox 1.

An example search string was (father* OR paternal OR “new dad*” OR “expecting father*” OR “father-to-be” OR “male partner*” OR “non-birthing parent*”) AND (“postnatal depression” OR “postpartum depression” OR “perinatal mental health” OR anxiety OR stress OR wellbeing OR “parenting stress” OR adjustment OR “help-seeking”) AND (digital OR online OR web-based OR internet OR eHealth OR mHealth OR “mobile app*” OR smartphone OR SMS OR “text message*” OR WhatsApp OR telehealth OR gamif* OR game* OR “serious game*” OR “game-based”) AND (intervention* OR program* OR therap* OR treatment OR support OR education OR psychoeducation OR feasibility OR acceptability OR usability OR implementation OR engagement OR evaluation).

These terms were searched across titles, abstracts, keywords, and full texts. The search string was adapted to the syntax and functionality of each database. To enhance the search strategy and refine the RQs, faculty librarians with expertise in systematic and scoping review methodology were consulted.

Textbox 1. **Summary and search terms.**

Population mental health and well-being

- Father*
- Paternal
- New dad
- Expecting father
- Father to be
- Nonbirthing parents
- Postnatal depression
- Postpartum depression
- Prenatal mental health
- Anxiety
- Stress
- Adjustment
- Psychological well-being
- Help-seeking
- Psychological support

Technology terms

- Digital
- Online
- Web-based
- Website
- Internet
- eHealth
- mHealth
- Mobile app*
- Smartphone
- Telehealth
- Gamif*
- Game*

Intervention or evaluation

- Intervention
- Program
- Therap*
- Treatment
- Support
- Education
- Psychoeducation
- Feasibility
- Acceptability
- Usability
- Implementation
- Evaluation

### Eligibility Criteria and Screening Process

This review focused on technology-based interventions targeting paternal perinatal mental health, and only papers that met the predefined inclusion and exclusion criteria were retained for analysis, as detailed in Textbox 2.

Textbox 2. **Inclusion and exclusion criteria.**

Inclusion criteria

- Full-text, peer-reviewed studies published in English.
- Fathers, expectant fathers, new fathers, male partners, or nonbirthing parents were active participants, target users, or explicitly reported as a relevant subgroup, including parent or couple interventions, where paternal or partner outcomes were reported.
- Studies addressed paternal PND, perinatal mental health, psychological well-being, help-seeking, parenting stress, adjustment to fatherhood, partner support, or closely related outcomes during the perinatal period.
- Studies described a digital, technology-based service pathway or support strategy.
- Studies reported intervention design, protocol details, feasibility, usability, acceptability, implementation, engagement, screening, referral, or relevant outcome data.

Exclusion criteria

- Fathers or nonbirthing parents were not active participants, target users, or a reported subgroup.
- Paternal mental health or well-being was not targeted or meaningfully addressed.
- Studies were not peer-reviewed or not available in English.
- The intervention was primarily focused on clinical simulation or professional training rather than support for fathers in community or home contexts.

A total of 3223 records were obtained from the search strategy listed in Textbox 1. All records were initially screened for duplicates by author 1, resulting in 82.2% (2649/3223) unique papers. Author 1 then screened all titles using the inclusion criteria, identifying 1.2% (32/2649) potentially relevant papers for the review. Because only 32 papers remained after title screening, the review team proceeded directly to full-text screening rather than conducting a separate abstract screening stage, allowing for a more thorough assessment of eligibility. Full texts were independently assessed by 2 reviewers against the predefined inclusion criteria, with author 1 identifying 11 papers for potential inclusion and author 2 identifying 6 (reviewer agreement 84%, Cohen [16] κ=0.61). Discrepancies were resolved through discussion between the 2 reviewers and arbitration by author 3, who has extensive experience in digital technologies in health application research.

Although initial agreement was moderate, both reviewers agreed on all 6 studies included. The discrepancies arose because author 1, newer to review methodology, identified 5 additional studies that did not meet full criteria. Following discussion with author 2 and author 3, who have more experience in digital health research, consensus was reached that these additional studies did not meet eligibility criteria, resulting in full agreement (100%) on the final 6 included studies.

### Data Extraction and Analysis

The following outlines the data extraction and analysis procedures for each RQ, corresponding to the structure of the *Results* section. Data were charted using a structured extraction form developed for this review. Extracted fields included author and year, country, study design, evidence type, population, sample size, intervention description, delivery mode, delivery format, delivery duration, digital component, theoretical or design basis, outcome measures, data collection method, key feasibility, acceptability, usability, engagement, or outcome findings. Data extraction was conducted by the first author, with uncertainties and interpretive decisions discussed collaboratively with the research team.

Findings were synthesized using a descriptive and comparative approach that focused on mapping the type and maturity of evidence, comparing delivery modes and formats, identifying how fathers were positioned within each study, summarizing measured outcomes, and identifying gaps in the evidence base. Consistent with the exploratory aims of a scoping review, and given the small number of studies and substantial heterogeneity across interventions, populations, and outcomes, a systematic review or meta-analysis was not considered appropriate for this evidence base.

## Results

**Figure 1.**
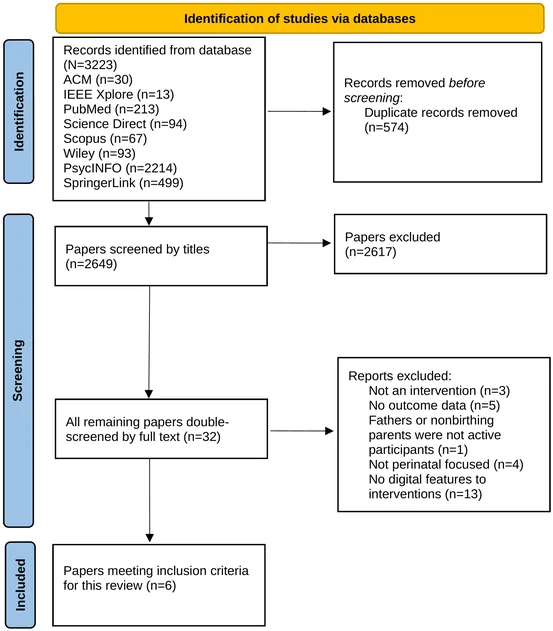
Screening and inclusion process.

Six studies met the eligibility criteria for this scoping review. They varied in design and methodological purposes, reflecting a heterogeneous evidence base characteristic of an early-stage, emerging field of research. Studies were published between 2017 and 2025 and were conducted across Australia, the United States, the Netherlands, Turkey, and Israel. Table 1 presents the characteristics of each study, and Figure 1 details the specific numbers from each database.

**Table 1.** Overview of the included studies.

Author and year	Fletcher et al [17] (2017)	Missler et al [18] (2020)	Canfield et al [19] (2023)	Karakoç and Kalkan [20] (2024)	Kaner et al [21] (2024)	Teague et al [11] (2025)
Country	Australia	Netherlands	United States	Turkey	Israel	Australia
Delivery mode	Smartphone-based SMS/text-message intervention	Hybrid psychoeducational intervention with online and digital components	Online web-based intervention	Hybrid postpartum education+digital follow-up	Online group intervention	Mobile health app intervention
Delivery format	Short SMS messages with embedded links and mood-tracking questions	Booklet, online video, prenatal home visit, and postnatal telephone call	Self-administered online CBT^a^-based Mothers and Babies Online Course, delivered through Qualtrics	Paternal support training delivered before postpartum discharge, with WhatsApp follow-up	Father-only online discussion groups led by a male facilitator	Prototype app, Rover, including chatbot, mood tracking, goal tracking, CBT/mindfulness exercises, resources, notifications, and gamification
Delivery duration	From 12 weeks of gestation to 24 weeks after birth	Delivered during late pregnancy and early postpartum, intervention included prenatal components and a postnatal phone call	8-week course; one lesson per week recommended	Initial training within the first 48 hours postpartum; follow-up to postpartum day 10	Six weekly sessions, each approximately 75 minutes	Fathers used the app for 4 weeks; clinicians evaluated it for 1 week
Methodology	Feasibility and acceptability study	Randomized controlled trial	Mixed methods pilot randomized controlled feasibility trial	Randomized controlled trial	Mixed methods randomized controlled trial	Feasibility, acceptability, and usability study using a design science approach
Population	Soon-to-be and new fathers	Pregnant women and partners/expecting parents	Pregnant people with elevated depression or anxiety symptoms and their cohabiting partners	Married postpartum couples/fathers and mothers	New partnered fathers with an infant under 1 year	Fathers with pregnant partners or infants under 12 months; mental health clinicians
Sample size	520 enrolled fathers	138 pregnant women and 96 partners	30 dads; 15 intervention and 15 controls	80 fathers randomized; data collected from 160 couples	122 fathers; 62 intervention and 60 comparison	43 fathers and 10 mental health clinicians
Intervention focus	Supporting fathers’ transition to fatherhood through father-infant connection, partner support, and fathers’ self-care	Preventing postpartum parenting stress, depression, and anxiety, improving parental well-being and caregiving quality	Reducing perinatal mood and anxiety symptoms through online CBT-based psychoeducation and partner-inclusive support	Increasing paternal support and improving dyadic adjustment, happiness, and psychological resilience	Supporting fatherhood transition, normalizing experiences, reducing loneliness, increasing awareness of fatherhood role, and supporting couple relationship	Father-specific perinatal mental health support through mood tracking, mindfulness, goal setting, CBT-informed exercises, chatbot support, and gamified app design
Outcome measures used	K6^b^^,^^c^, completion/retention, link-click engagement, mood-tracker responses, and poststudy feedback survey	Parenting Stress Index items^b^, EPDS^b^^,^^d^, HADS^e^ anxiety^b^, parental well-being, parenting self-efficacy, satisfaction with parenting role, sleep, parent-infant bonding, breastfeeding duration, room-sharing, infant crying/feeding/sleeping problems, and intervention usefulness	PHQ-9^b^^,^^f^ for eligibility/screening, GAD-7^b^^,^^g^, EPDS^b^, retention, adherence analytics, satisfaction, and acceptability interviews	Revised Dyadic Adjustment Scale^b^, Short Form Oxford Happiness Questionnaire^b^, and Brief Resilience Scale^b^	EPDS^b^, Israeli Marital Quality Scale^b^, attendance rate, session transcripts, and 2-year follow-up feedback questionnaire	EPDS^b^, DASS-21^h^ anxiety and stress subscales^b^, System Usability Scale^b^; Mobile App Rating Scale^b^, Mobile Agnew Relationship Measure^b^, app usage statistics, and qualitative feedback
How outcomes were collected	Automated program data, including completion, link clicks, and mood-tracker responses; K6 screening; poststudy survey	Online questionnaires sent to mothers and fathers, intervention uptake/usefulness ratings, and postpartum follow-up assessments	Online surveys at baseline, 4 weeks, and 8 weeks; eMB^i^ platform analytics; postintervention interviews via Zoom (Zoom Communications, Inc)	Self-report questionnaires collected from mothers and fathers at pretest within 48 hours after birth and posttest on postpartum day 10	Quantitative self-report questionnaires, attendance records, transcripts of closing group sessions, and email feedback questionnaire 2 years after the intervention	Baseline and follow-up surveys, app usage analytics, validated usability/app-quality/therapeutic-alliance scales, and open-ended qualitative feedback

a CBT: cognitive behavioral therapy.

b Validated measure or validated scale/subscale.

c K6: Kessler Psychological Distress Scale-6.

d EPDS: Edinburgh Postnatal Depression Scale.

e HADS: Hospital Anxiety and Depression Scale.

f PHQ-9: Patient Health Questionnaire-9.

g GAD-7: Generalized Anxiety Disorder-7.

h DASS-21: Depression Anxiety Stress Scales-21.

i eMB: electronic mothers and babies course.

The first RQ examined the types of evidence available and how studies varied by delivery mode, methodology, and population. Delivery modes varied substantially, including SMS messaging delivered to fathers’ smartphones [17], WhatsApp-supported follow-up [20], online video within a multicomponent intervention [18], an asynchronous online cognitive behavioral therapy (CBT)–based course [19], synchronous online father-only groups [21], and a prototype mHealth app, called the Rover app [11]. Methodologically, the studies varied in the extent to which digital delivery was central to the intervention. In the SMS4dads feasibility study [17], the digital component was the main intervention mechanism, delivered through text messages to fathers’ smartphones. Similarly, the Rover app study [11] placed digital design at the center of the intervention by evaluating a father-specific mHealth app. The online group intervention [21] and online CBT-based dyadic intervention [19] also used digital delivery as the primary access pathway. In contrast, the psychoeducational intervention [18] and paternal support training [20] used digital elements as supplementary components, such as online video, telephone follow-up, or WhatsApp follow-up. The target populations also varied. Several studies were explicitly father-focused, including SMS4dads, the online father group intervention, and the Rover mHealth app [11,17,21], while other studies included fathers or partners within couple, dyadic, or expectant-parent interventions [18-20].

The second RQ examined how the interventions were characterized in terms of delivery mode, format, duration, and intervention components. The interventions can be grouped into 3 delivery models: fully digital, online group-based, and hybrid interventions with digital reinforcement. The fully digital interventions, SMS4dads [17] and the Rover mHealth app [11], were accessed independently of face-to-face health services. SMS4dads delivered stage-based text messages and embedded links across pregnancy and early fatherhood, while Rover offered app-based features such as mood tracking, goal tracking, mindfulness exercises, chatbot interaction, resources, and gamified elements.

The online group [21] and online CBT-based [19] intervention used digital delivery to create structured support spaces. The former comprised 6 weekly father-only group sessions led by a male facilitator, focused on peer normalization and fatherhood identity; the latter was an 8-week structured course for pregnant people and partners targeting perinatal mood and anxiety symptoms.

The hybrid interventions [18,20] used digital components to reinforce nondigital support. The psychoeducational intervention [18] combined a booklet, online video, prenatal home visit, and postnatal telephone call, while the paternal support training [20] included WhatsApp follow-up after an initial in-person session.

Duration also varied: SMS4dads [17] spanned from pregnancy to postpartum; Rover [11] was evaluated over 4 weeks, the online group intervention [21] ran 6 weeks, the CBT course [19] ran 8 weeks, and the hybrid interventions [18,20] involved shorter, more targeted contact around late pregnancy and early postpartum.

The third RQ examined what outcomes have been measured across studies and what gaps remain in the evidence base. All 6 studies reported completed intervention, feasibility, acceptability, usability, or outcome evaluations [11,17-21]. Across the included studies, outcome measurement clustered around 4 broad domains: mental health symptoms, relational or dyadic functioning, feasibility and engagement, and acceptability or usability. Mental health symptoms were the most commonly measured domain. Five studies included measures of psychological distress, depression, anxiety, stress, or parenting stress [11,17-19,21]. The Edinburgh Postnatal Depression Scale (EPDS) was the most frequently used depression-related measure, appearing across several studies. Other symptom measures included the Kessler Psychological Distress Scale-6, Patient Health Questionnaire-9, Generalized Anxiety Disorder-7, Hospital Anxiety and Depression Scale anxiety subscale, and Depression Anxiety Stress Scales-21 anxiety and stress subscales.

Relational, dyadic, and family-functioning outcomes were also represented, but they were measured inconsistently. Some studies assessed dyadic adjustment, marital quality, partner relationship quality, happiness, psychological resilience, parenting self-efficacy, parent-infant bonding, or caregiving quality [18,20,21].

Feasibility and engagement were measured in several studies [11,17,19,21], particularly those evaluating digital or app-based interventions. SMS4dads reported completion, retention, link-click engagement, and responses to mood-tracking questions. The online CBT-based intervention collected adherence data through platform analytics, while the Rover mHealth app collected app usage statistics. The online father group intervention reported attendance rates, and other studies collected data on intervention usefulness or feedback.

Acceptability, usability, and user experience were also commonly examined, but mainly in studies where the digital component was central to the intervention. The Rover app study used validated usability and app-quality measures, including the System Usability Scale, Mobile App Rating Scale, and Mobile Agnew Relationship Measure, alongside qualitative feedback [11]. SMS4dads [17] and the online CBT-based intervention [19] also collected postintervention feedback or acceptability data. Other hybrid studies assessed perceived usefulness or qualitative experiences [18,20,21].

Several gaps were evident in outcome measurement across the included studies. Father-specific outcomes, such as paternal help-seeking intention, stigma, paternal self-efficacy, or engagement with professional support pathways, were rarely measured; most studies instead relied on general mental health, relational, or usability measures. In studies where fathers were included within broader couple-based or expectant-parent interventions, paternal outcomes were not always reported separately from maternal or dyadic outcomes. Most studies relied on self-report measures, and follow-up periods were generally short, with no studies reporting outcomes beyond the early postpartum period.

## Discussion

### Principal Findings

This scoping review identified 6 studies examining technology-based, digitally delivered, or hybrid interventions relevant to paternal perinatal mental health. Overall, the evidence is best understood as an emerging intervention landscape rather than a mature effectiveness literature. Several studies focused primarily on feasibility, acceptability, usability, engagement, or intervention design, while others reported early outcome evaluation. This indicates that the field is beginning to move beyond protocol-level intervention development, but it has not yet produced a consistent or robust evidence base from which firm conclusions about effectiveness can be drawn.

A key distinction concerns the role of digital delivery within the intervention model. Because digital tools functioned as the primary support mechanism in some studies and as only a supplementary component in others, the effectiveness of the digital component cannot be interpreted uniformly. In hybrid interventions, observed outcomes may reflect the combined effects of face-to-face, printed, professional, relational, and digital components, rather than the independent contribution of digital delivery itself.

The interventions also differed in design logic, ranging from brief, low-intensity, repeated contact (such as SMS prompts and mood-tracking check-ins) to more structured formats (such as a multiweek course or a multifeature app), with Rover [11] representing the clearest example of a father-specific digital design. These differences suggest that digital technology is not being used in a single consistent way across the literature; rather, it functions variously as a delivery platform, engagement tool, follow-up mechanism, self-monitoring system, or design feature.

Outcome measurement varied considerably across the 6 studies. Mental health symptoms were the most commonly measured domain, with several studies using validated measures such as the EPDS, Kessler Psychological Distress Scale-6, Patient Health Questionnaire-9, Generalized Anxiety Disorder-7, Hospital Anxiety and Depression Scale, or Depression Anxiety Stress Scales-21. This suggests partial convergence around depression and psychological distress measurement, particularly through the EPDS. However, there was no shared outcome framework across studies. Relational and adjustment-related outcomes, including dyadic adjustment, marital quality, psychological resilience, parenting self-efficacy, caregiving quality, and parent-infant bonding, were measured inconsistently and often reflected the specific focus of each intervention. Engagement outcomes were also operationalized differently, including attendance, retention, link clicks, app interactions, mood-tracker responses, lesson adherence, and qualitative feedback. This variation limits comparison across studies and makes it difficult to identify which intervention formats or components are associated with stronger engagement or more meaningful outcomes.

Across the evidence base, findings were more consistent for feasibility, acceptability, usability, and engagement than for clinical effectiveness. SMS delivery, online group discussion, mobile app features, brief digital contact, and digital follow-up were generally reported as acceptable or usable by participants. Features such as father-specific messaging, humor, baby-centered content, mood tracking, mindfulness activities, goal setting, peer normalization, and male facilitation appeared to support engagement in some studies. However, these features were not systematically evaluated across all included studies, and positive acceptability should not be interpreted as evidence of effectiveness. While several studies reported promising engagement or user experience findings, evidence that these interventions produce sustained behavioral change, improved help-seeking, or reduced paternal perinatal depression remains limited.

Effectiveness findings were mixed and should be interpreted cautiously. Some studies reported improvements or protective effects in relational and well-being–related outcomes, such as dyadic adjustment, happiness, psychological resilience, marital quality, or perceived social connection. However, effects on depression, anxiety, parenting stress, and other mental health outcomes were less consistent. In addition, several studies measured paternal mental health indirectly or as a secondary outcome, and follow-up periods were generally short. This limits the extent to which conclusions can be drawn about whether technology-based or hybrid interventions specifically reduce paternal perinatal depression.

Included studies also varied in the extent to which they targeted father-specific outcomes, such as paternal help-seeking, stigma reduction, paternal self-efficacy, parenting confidence, or engagement with professionals, which limits the interpretation of relevance to fathers; a literature gap addressed further in the *Limitations* section below.

### Implications for Digital Intervention Design

The gaps identified in this scoping review point to several priorities for future research. Most pressing is the need for primary studies designed specifically to address paternal perinatal mental health, rather than positioning fathers as secondary participants in mother-focused or general parent-focused interventions. Father-specific interventions should explicitly address fathers’ mental health needs, barriers to help-seeking, stigma, paternal identity transition, partner communication, and father-infant bonding.

Future studies should also articulate a clear theoretical basis for intervention design. At present, the included studies vary in whether and how they describe theoretical foundations or mechanisms of change. Clearer theoretical grounding would allow comparison across interventions and help identify which components are most likely to influence outcomes such as mental health literacy, help-seeking intention, emotional regulation, relational support, and engagement with professional care. In addition, future studies should use validated, father-specific outcome measures where possible and have longer follow-up periods to determine whether any benefits are sustained beyond the immediate postpartum period.

A promising avenue for future research includes serious games and gamified interventions. Although 1 included study incorporated gamification elements, no study evaluated a fully developed serious game for paternal perinatal mental health. Game-based approaches may offer novel ways to support mental health literacy, emotional reflection, coping practice, help-seeking attitudes, partner communication, and a means of addressing known barriers to fathers’ help-seeking such as time and stigma.

### Limitations of the Included Studies

The included studies have several limitations that affect interpretation of this review. First, although the updated search identified 6 studies, the evidence base remains small and heterogeneous, limiting direct comparison across studies and preventing firm conclusions about the effectiveness, scalability, or suitability of digital interventions for paternal perinatal depression.

Second, not all included studies positioned fathers’ postnatal mental health as the primary intervention target. Some studies were designed specifically for fathers, whereas others included fathers or partners within broader couple-based or expectant-parent interventions. While these studies are relevant to paternal postnatal mental health, they do not always isolate paternal outcomes or address fathers’ unique barriers to help-seeking, stigma, masculine norms, fatherhood identity, or father-infant bonding. As a result, conclusions about father-specific digital intervention design should be interpreted with caution.

Third, most studies relied heavily on self-report data and short follow-up periods. While self-report measures are appropriate for assessing perceived symptoms, usability, and acceptability, they may be affected by recall bias, social desirability bias, or participant expectations. The limited duration of follow-up also means that little is known about whether digital interventions can produce sustained changes in paternal mental health, help-seeking behavior, relationship functioning, or parenting confidence beyond the early postpartum period.

Fourth, the diversity and representativeness of participants were limited across the included evidence. Several studies used relatively small, educated, partnered, or culturally homogeneous samples. This restricts the generalizability of findings to fathers from diverse cultural, socioeconomic, geographic, linguistic, or family-structure backgrounds. Further research is needed to understand whether digital interventions are acceptable, accessible, and relevant for fathers who may experience additional barriers to support, including fathers in low-resource settings, nonresidential fathers, same-sex parents, and fathers from culturally and linguistically diverse communities.

### Limitations of This Review

Although the search strategy was broad to capture digital, online, mobile, SMS-based, and game-informed interventions, relevant studies may still have been missed due to variation in terminology across digital health, perinatal mental health, and fatherhood research. The review was limited to peer-reviewed English-language publications, which may have excluded relevant gray literature, non-English studies, service evaluations, or unpublished digital health projects.

The search did not include CINAHL or Web of Science in the database searches due to resource constraints, both of which index nursing, allied health, and interdisciplinary literature. Given the overlap between these databases and those searched, it is unlikely that a substantial amount of relevant literature was missed; however, some relevant studies may have been overlooked as a result. Future reviews could include broader database coverage to minimize the risk of omission.

In addition, title screening was conducted by a single reviewer, which may have introduced selection bias, although later stages of screening involved independent review and consensus discussion. Consistent with scoping review methodology, this review aimed to map the available evidence rather than formally appraise study quality or pool effect sizes. Therefore, the findings should be interpreted as a descriptive and interpretive overview of an emerging evidence base, rather than as evidence of clinical effectiveness.

### Conclusion and Future Work

While the limited evidence base precludes definitive conclusions, the 6 included studies tentatively suggest that digital modalities may offer feasible and acceptable ways of engaging some fathers and partners during the perinatal period. Digital modalities may help reduce father-specific barriers related to time, geography, stigma, and reluctance to attend face-to-face services; however, future research is required to systematically evaluate and confirm these potential advantages.

Intervention features such as father-specific messaging, humor, baby-centered content, brief digital contact, mood tracking, goal setting, mindfulness activities, and peer normalization were described as potentially supportive of engagement. However, the evidence that these features translate into sustained behavior change, improved help-seeking, or reduced paternal PND remains limited. Acceptability and usability findings should therefore be understood as early design evidence that still requires proof of clinical effectiveness.

The current evidence base remains too small and heterogeneous to support firm conclusions about the scalability, suitability, or effectiveness of digital interventions for fathers experiencing perinatal mental health challenges. Future research should prioritize father-specific digital interventions that are theory-informed, co-designed with fathers, and evaluated using rigorous methods, including validated father-specific outcomes, longer follow-up periods, transparent reporting of engagement and attrition, and more diverse participant samples.

Fathers remain a consistently underserved population despite the prevalence of paternal perinatal mental health challenges. Digital interventions offer a promising and timely avenue for future research, with the potential for innovative, father-tailored solutions that could meaningfully advance both clinical practice and public health initiatives in this critical area.

## Supplementary material

10.2196/95238 Checklist 1 PRISMA-ScR checklist.

## Abbreviations

CBT: cognitive behavioral therapy
EPDS: Edinburgh Postnatal Depression Scale
mHealth: mobile health
PND: postnatal depression
PRISMA-ScR: Preferred Reporting Items for Systematic Reviews and Meta-Analyses Extension for Scoping Reviews
RQ: research question
